# A randomised controlled trial of a mitochondrial therapeutic target for bipolar depression: mitochondrial agents, *N*-acetylcysteine, and placebo

**DOI:** 10.1186/s12916-019-1257-1

**Published:** 2019-01-25

**Authors:** Michael Berk, Alyna Turner, Gin S. Malhi, Chee Ng, Susan M. Cotton, Seetal Dodd, Yuval Samuni, Michelle Tanious, Claire McAulay, Nathan Dowling, Jerome Sarris, Lauren Owen, Astrid Waterdrinker, Deidre Smith, Olivia M. Dean

**Affiliations:** 1 IMPACT Strategic Research Centre, School of Medicine, Barwon Health, Deakin University, P.O. Box 291, Geelong, VIC Australia; 2 Department of Psychiatry, University of Melbourne, Royal Melbourne Hospital, Level 1 North, Main Block, Parkville, VIC Australia; 3 Florey Institute for Neuroscience and Mental Health, University of Melbourne, Kenneth Myer Building, 30 Royal Parade, Parkville, VIC Australia; 4 Orygen, The National Centre of Excellence in Youth Mental Health, 35 Poplar Rd, Parkville, VIC Australia; 5 School of Medicine and Public Health, Faculty of Health and Medicine, the University of Newcastle, Callaghan, NSW Australia; 6 CADE Clinic, Royal North Shore Hospital, Northern Sydney Local Health District, St Leonards, NSW Australia; 7 Academic Department of Psychiatry, Kolling Institute, Northern Sydney Local Health District, St Leonards, NSW Australia; 8 ARCHI, Sydney Medical School Northern, University of Sydney, Sydney, NSW Australia; 9 Department of Psychiatry, University of Melbourne, the Melbourne Clinic, 130 Church St Richmond, Melbourne, VIC Australia; 10 Centre for Youth Mental Health, The University of Melbourne, 35 Poplar Rd, Parkville, VIC Australia; 11 NICM, School of Health and Science, Western Sydney University, Campbelltown, NSW Australia; 12 School of Psychology, University of Central Lancashire, Preston, UK; 13 Melbourne Health, 300 Grattan St, Melbourne, VIC Australia

**Keywords:** Mitochondria, Bipolar disorder, Depression, Nutraceutical, Adjunctive, Oxidative stress

## Abstract

**Background:**

A phasic dysregulation of mitochondrial bioenergetics may operate in bipolar disorder, increased in mania and decreased in depression. We aimed to examine efficacy of two add-on treatments in bipolar depression: *N*-acetylcysteine (NAC) and NAC with a combination of nutraceutical agents that may increase mitochondrial biogenesis.

**Methods:**

A three-arm 16-week, double-blind, randomised, placebo-controlled trial, adjunctive to usual treatment, was conducted. Participants (*n* = 181) with bipolar disorder and current depressive symptoms were randomised to 2000 mg/day NAC (*n* = 59), 2000 mg/day NAC with the combination nutraceutical treatment (CT, *n* = 61), or placebo (*n* = 61). The primary outcome was change in Montgomery-Åsberg Depression Rating Scale (MADRS) total score from baseline to week 16. Young Mania Rating Scale, Clinical Global Impression (CGI)-Improvement and CGI-Severity scales, Patient Global Impression scale, Social and Occupational Functioning Assessment Scale (SOFAS), Longitudinal Interval Follow-Up Evaluation - Range of Impaired Functioning Tool (LIFE-RIFT), and Quality of Life Enjoyment, and Satisfaction Questionnaire Short Form (Q-LES-Q-SF) were secondary outcomes.

**Results:**

One hundred forty-eight participants had post-randomisation data and were analysed (NAC = 52, CT = 47, Placebo = 49). No between-group differences were found for the rate of change between baseline and 16 weeks on any of the clinical and functioning variables. Improvements in MADRS, BDRS, SOFAS, and LIFE-RIFT scores from baseline to the week 20 post-discontinuation visit were significantly greater in the CT group compared to those in the placebo. At week 20, the CGI-I was significantly lower in the CT group versus placebo. Gastrointestinal symptoms were significantly greater in the NAC than in the placebo group.

**Conclusions:**

These overall negative results, with no significant differences between groups detected at the primary outcome but some positive secondary signals, suggest either delayed benefit of the combination or an improvement of symptoms on withdrawal which warrants further exploration regarding the composition, mechanisms, and application of mitochondrial agents in illnesses characterised by mitochondrial dysfunction.

**Trial registration:**

ANZCTR (ACTRN12612000830897).

## Background

The pathophysiology of bipolar disorder remains uncertain, but there is evidence of a critical role of mitochondrial dysfunction [1]. Phenotypically, bipolar disorder is a biphasic disorder of energy [2], whereby energy is increased in mania and decreased in the depressive phase. Both mitochondrial respiration and adenosine triphosphate production seem increased in bipolar mania, while mitochondrial function appears reduced in the depressive or euthymic phase of the disorder [3–5]. It can be postulated that bipolar disorder involves a phasic dysregulation of mitochondrial bioenergetics, characterised by inability to upregulate biogenesis in response to metabolic demands in depression, and to downregulate in mania [5]. The precise source of this phasic dysregulation remains uncertain, although many operative biological elements are implicated [4].

Interventions that enhance mitochondrial function may have the potential to reduce depressive symptoms in bipolar disorder. Therefore, the aim of this three-arm study was to examine the efficacy in bipolar depression of two add-on treatments: *N*-acetylcysteine (NAC) 2000 mg/day, which has been shown to have potential antidepressant and mitochondrial biogenesis effects, and NAC 2000 mg/day together with a “cocktail” of nutrient agents (a combination nutraceutical treatment [CT] of a total of 16 compounds that have potential efficacy in increasing mitochondrial biogenesis). The key elements of the combination comprised acetyl l-carnitine (ALC), ubiquinone (coenzyme Q10), and alpha lipoic acid (ALA), in addition to co-factors involved in mitochondrial function. The rationale for this combination has been explicated in detail in a published protocol [6].

We hypothesised that compared to the placebo, both adjunctive treatments would improve symptoms of depression, based on a change from baseline to week 16 (primary endpoint) in the total score on the Montgomery-Åsberg Depressive Rating Scale (MADRS) as the primary outcome measure. Secondary outcomes included changes from baseline to week 16 and week 20 (4-week post-discontinuation) in the score of overall symptom severity and improvement (clinician and participant rated), quality of life, functional impairment, and symptoms of mania and anxiety.

## Method

### Study overview

The study was a 16-week, multi-site, randomised, double-blind, parallel group trial of a CT, NAC, or placebo in the depressive phase of bipolar disorder. Participants received 16 weeks of daily adjunctive treatment with assessment visits at baseline and 4, 8, 12, 16 (primary endpoint), and 20 weeks (4-week post-discontinuation). A phone interview at week 2 was conducted to promote adherence and record any adverse events. Participants were assigned randomly and consecutively to treatment with CT, NAC, or placebo (1:1:1 design) in a double-blind fashion. All participants remained on treatment as usual (pharmaceutical and complimentary) for the duration of the trial. The trial was conducted according to the Good Clinical Practice guidelines. Research ethics committee approval was obtained at all participating sites (Barwon Health, The Geelong Clinic, Royal North Shore Hospital, and The Melbourne Clinic Human Research and Ethics Committees). The study was registered on the Australian New Zealand Clinical Trials Registry (ACTRN12612000830897).

### Randomisation and masking

Participant number allocation to treatment arm was randomly assigned using permutated block randomisation. The computer-generated randomisation plan was developed by an independent researcher utilising four-to-a-block design. Participant numbers were sequentially allocated by trial clinicians. To facilitate the double-blinding process, the trial medications (CT, NAC only, and placebo) were packed in the medicopacks and dispensed by an independent pharmacist in sealed containers. Medicopacks and capsules in all arms were identical, to conceal treatment allocation and blinding. The consultant statistician (SC), investigators, and participants were blinded to the group allocation. Participants were informed and unblinded via letters sent at the completion of the full study.

### Investigational products

All components of the active treatment arms are well-tolerated by humans at the doses proposed in this study and are currently available for purchase without prescription in the USA and Australia. The CT comprised *N*-acetylcysteine (NAC) 2000 mg, acetyl l-carnitine (ALC) 1000 mg, ubiquinone (Co Q10) 200 mg, magnesium (as orotate 500 mg) 64 mg, calcium ascorbate dehydrate (equiv ascorbic acid 200 mg) 242 mg, cholecalciferol (equiv vitamin D3 250 IU) 12.5μg, α-tocopherol (equiv natural vitamin E 50 IU) 60 IU, alpha lipoic acid (ALA) 150 mg, Retinyl palmitate (equiv vitamin A 3000 IU) 900ugREIU, and vitamin B co-factors: biotin (vitamin H) (600μg), thiamin hydrochloride (100 mg), riboflavin (100 mg), nicotinamide (200 mg), calcium pantothenate (100 mg), pyridoxine hydrochloride (100 mg), folic acid (800 μg), and cyanocobalamin (vitamin B12) (800 μg). They were given in the form of five capsules twice a day, including as follows: four 500 mg NAC capsules, two twice per day; two 500 mg ALC capsules, one twice per day; two capsules with 75 mg Coenzyme Q10, 75 mg ALA, and 32 mg magnesium, one twice per day; and two capsules with 25 mg Coenzyme Q10, and vitamins E, C, A, D3, H, and B, one twice per day. The other active arm consisted of 2000 mg of NAC as per the CT arm, in addition to placebo capsules matched for the remaining CT active capsules. The placebo arm had matched product for all active capsules, given in a double-dummy design.

All investigational products were produced under Pharmaceutical Good Manufacturing Practice. The NAC and NAC placebo were supplied by Nutrition Care, Australia. The additional nutraceutical components of the CT were supplied by BioCeuticals, Australia, with matching placebos manufactured by Catalent, Australia.

### Recruitment procedure

Participants were recruited through their treating clinicians and case managers, and self-referral via advertisements (newspaper, online, radio, media via press releases, and flyers at relevant health and community locations). Potential participants were contacted and briefly screened, and a preliminary interview was scheduled. All participants provided written informed consent before enrolment.

### Inclusion criteria

Participants (aged 18 years and over) meeting Diagnostic and Statistical Manual of Mental Disorders IV-TR criteria for bipolar disorder (I, II, or not otherwise specified) on a structured clinical interview (Mini-International Neuropsychiatric InterviewPlus) with a current acute depressive episode (MADRS score ≥ 20) were recruited. In addition, participants must have had the capacity to consent to the study and comply with study procedures and be using effective contraception if female, sexually active, and of childbearing age. Participants currently under any form of therapy needed to remain on stable therapy for at least 1 month prior to randomisation without significant adjustments to dose. Participants were required to nominate a current treating physician. If there was a delay of > 7 days between screening and baseline (randomisation) assessments, or randomisation and medication commencement, the MADRS was readministered to ensure the participant still met eligibility criteria (≥ 20).

### Exclusion criteria

Exclusion criteria included the following: participants with a known or suspected active systemic medical disorder, recent gastrointestinal ulcers, epilepsy or renal stones, pregnancy or lactation, or currently taking > 250 mg of NAC, > 250 mg of ALC, > 25 mg of coenzyme Q10, or > 200μg of selenium/day (a 1-month washout period was required if participants were taking these study preparations). Participants currently enrolled in any other intervention study were excluded. Individuals treated with warfarin or phenytoin or individuals who were intolerant to or had an allergic reaction to any components of the preparation were also excluded.

### Withdrawal procedure

Withdrawal from the trial occurred if the participants ceased taking their trial medication for seven consecutive days, ceased effective contraception, or became pregnant. Dose changes to existing medications or therapies, or addition or removal of an agent was accepted, and participants were allowed to continue with the trial. Participants were withdrawn from the study if they withdrew consent or at the discretion of the researcher given adverse events or loss to follow-up.

### Measurements

The participants were assessed at baseline using a structured clinical interview, the Mini-International Neuropsychiatric Interview Plus Version 5.0.0. A set of validated outcome measures was completed at baseline and weeks 4, 8, 12, 16, and 20 (4 weeks after treatment discontinuation). These measures included the MADRS (primary outcome), Hamilton Anxiety Rating Scale (HAM-A), Bipolar Depression Rating Scale (BDRS), Young Mania Rating Scale (YMRS), Clinical Global Impression (CGI)-Improvement and CGI-Severity scales, Patient Global Impression scale (PGI-I), Social and Occupational Functioning Assessment Scale (SOFAS), Longitudinal Interval Follow-Up Evaluation - Range of Impaired Functioning Tool (LIFE-RIFT), and Quality of Life Enjoyment and Satisfaction Questionnaire Short Form (Q-LES-Q-SF). Demographic data was collected including substance use, age, weight, height, sex, psychiatric history, and duration of illness. Adherence was monitored using capsule counts of returned clinical trial material. Adverse effects were recorded, intervened according to medical assessment, and monitored.

### Training

Raters had to complete a comprehensive certification process. All potential raters were required to view the overview training modules and complete annual reliability and inter-rater reliability assessments.

### Trial sites

The trial sites included Barwon Health and The Geelong Clinic in Geelong, The Melbourne Clinic in Melbourne, and the University of Sydney CADE Clinic based at Royal North Shore Hospital in Sydney. Recruitment was funded to take place between March 2013 and August 2015.

### Follow-up of adverse events

The research clinician followed up any participant who completed/withdrew from the study and had an ongoing adverse event 30 days after trial medication was ceased. If a participant withdrew or were withdrawn early from the study, they were requested to attend a final face-to-face interview, scheduled to occur as soon as possible to capture mood symptoms. If the participant was experiencing an adverse event at that time, they were arranged to be phoned 30 days later to follow up that event. If the participant did not attend a final face-to-face visit upon withdrawal, verbal permission was sought for a final monitoring phone call (30 days post-discontinuation). Participants who had an ongoing adverse event that was concerning them were asked to contact their general practitioner or treating physician.

### Statistical power

Overall power to detect significant differences was between the actual patterns of the means. Assuming a correlation of post-treatment scores with baseline measurements of 0.70 and an effect of the dosage such that either group (NAC and CT) differs from the placebo by 0.75 standard deviations, power will be maintained above 90% with 75 subjects in each group. Pairwise comparisons with 75 subjects per group in the three groups would enable effects smaller than 0.6 standard deviations to be detected with power of 80%. These effect sizes are in the small to moderate range. The experiment would thus be capable of detecting differences between groups of clinical and scientific interest with a total sample size of 225.

### Data analysis

The baseline characteristics of the cohort were examined using basic descriptive statistics such as means and standard deviations for continuous measures and frequencies and percentages for categorical variables. To determine differences between completers (data collected at more than one post-randomisation time point) and non-completers (no post-randomisation data), a series of independent samples *t* tests and chi-square (*χ*^2^) analyses were conducted.

For the analysis of primary and secondary outcomes, a modified intent-to-treat approach (mITT) was used with analysis on those with post-randomisation data. For the analysis of the primary outcome of severity of depressive symptoms (MADRS), a mixed model repeated measures (MMRM) was used. Within this model, the fixed effects were group (Placebo, NAC, CT), time (0, 4, 8, 12, 16, and 20 weeks), and site (Geelong, Melbourne, Sydney). The interaction between group and time was also examined. The MMRM includes a random intercept and slope over time. The relationships between observations across the six time points were modelled using unstructured covariance matrix. For the primary analyses, the focus was on planned comparisons that determined whether the rate of change from baseline to 16 weeks (primary end point at end of treatment) and from baseline to 20 weeks (post-discontinuation) differed among the groups. The planned comparisons were done to compare a treatment group (either NAC or CT) against the placebo; there was no comparison of the two active treatment groups as the focus was on exploring the benefits of both treatments independently versus the placebo. To examine group differences on the CGI-Improvement (CGI-I) and PGI-I at 16 and 20 weeks, one-way analysis of variance models were conducted, followed by two planned comparisons comparing the two active treatments to the placebo group. To examine differences between the groups with respect to adverse events, chi-square (*χ*^2^) analysis was conducted; significant *χ*^2^ values were followed by Bonferroni post hoc comparisons for proportions.

## Results

### Sample characteristics

Participants (*N* = 181) who met inclusion criteria were randomised to one of the three groups: *n* = 59 in the NAC group, *n* = 61 in the CT group, and *n* = 61 in the placebo group. 58.6% (*n* = 106) had a diagnosis of bipolar disorder I (BDI) and 41.4% (*n* = 75) had bipolar disorder II; 60.7% (*n* = 37) of each of the CT and placebo groups had a diagnosis of BDI whereas 54.2% (*n* = 37) in the NAC group had a diagnosis of BDI. 38.7% (*n* = 70) were recruited at the Melbourne site, 34.8% (*n* = 63) at the Sydney site, and 26.5% (*n* = 48) were recruited from Geelong. The baseline characteristics of this cohort are shown in Table 1. Participants were aged between 19.6 and 72.0 years (*M =* 45.5, *SD* = 12.3). The majority were female (63.5%, *n* = 115) and were not married or in a de facto relationship (54.1%, *n* = 98).

**Table 1:**
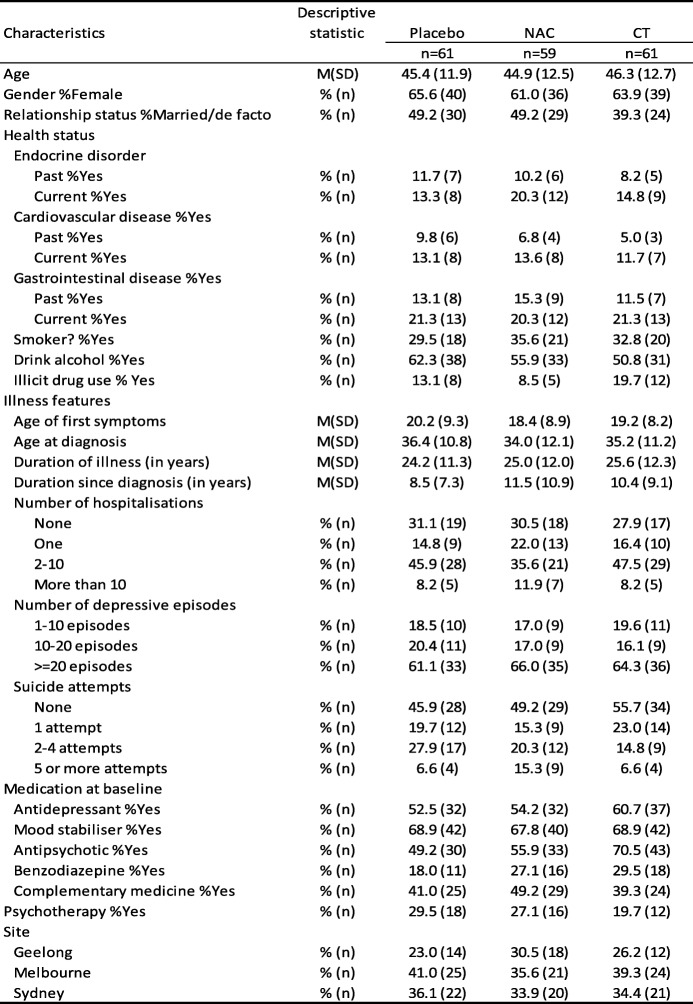
Descriptive statistics describing the baseline demographic and illness features for the three groups

Note: NAC, N-acetyl cysteine; CT, combination nutraceutical treatment

With respect to the physical health, 16.1% (*n* = 29) reported having a current endocrine problem, 12.8% (*n* = 23) have a current cardiovascular problem, and gastrointestinal problems were reported in 21.0% (*n* = 38) of the cohort. Approximately a third of the cohort currently smoked (32.6%, *n* = 59), and 56.4% (*n* = 102) consumed alcohol on a regular basis. The rate of alcohol use disorders (abuse or dependence) was 42.0% (*n* = 76). Current illicit drug use was reported in 13.8% (*n* = 25).

The average age of first symptoms was 19.3 years (*SD* = 8.8), and the average age of formal diagnosis was at 35.2 years (*SD* = 11.3). The mean duration of illness was 24.9 years (*SD* = 11.8), with the majority (43.1%, *n* = 78) having had between 2 and 10 hospital admissions. Most participants (63.8%, *n* = 104) had experienced 20 or more lifetime depressive episodes.

The medications prescribed at baseline included mood stabilisers (68.5%, *n* = 124), antipsychotics (58.6%, *n* = 106), antidepressants (55.8%, *n* = 101), and benzodiazepines (24.9%, *n* = 45). Regarding baseline mood stabiliser status, combining the three medications, 93.4% (*n* = 169) were on at least one of the medication types, lithium, mood stabiliser, and/or antipsychotic. There were no differences between the groups: placebo 88.5% *n* = 54, NAC 94.9% *n* = 56, and CT 96.7% *n* = 59. Other nutraceuticals (nutrient or herbal medicines) had been used by 43.1% (*n* = 78) of the cohort. About a quarter of the cohort had regular psychotherapy with a psychologist or a psychiatrist. The cohort had moderate levels of depression and anxiety, overall psychopathology, and moderate-severe impairment in social and occupational functioning (details are shown in Table 2). The most commonly reported comorbidities were social anxiety disorder, obsessive compulsive disorder, and panic disorder.

**Table 2:**
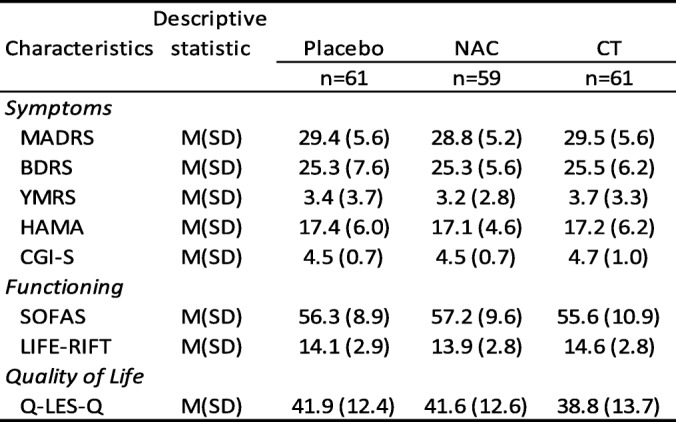
Baseline clinical characteristics for the three groups

Note: NAC, N-acetyl cysteine; CT, combination nutraceutical treatment; MADRS, Montgomery-Åsberg Depression Rating Scale; BDRS, Bipolar Depression Rating Scale; YMRS, Young Mania Rating Scale; HAM-A, Hamilton Anxiety Rating Scale; CGI-S Clinical Global Impressions - Severity Scale; ; SOFAS, Social and Occupational Assessment Scale; LIFE-RIFT, The Range of Impaired Functioning Tool; Q-LES-Q, Quality of Life

There were no significant differences between the placebo and the two treatment arms on any of the demographic characteristics detailed in Table 1. There were no differences noted on any of the clinical measures depicted in Table 2.

#### Completer analysis

**Fig. 1:**
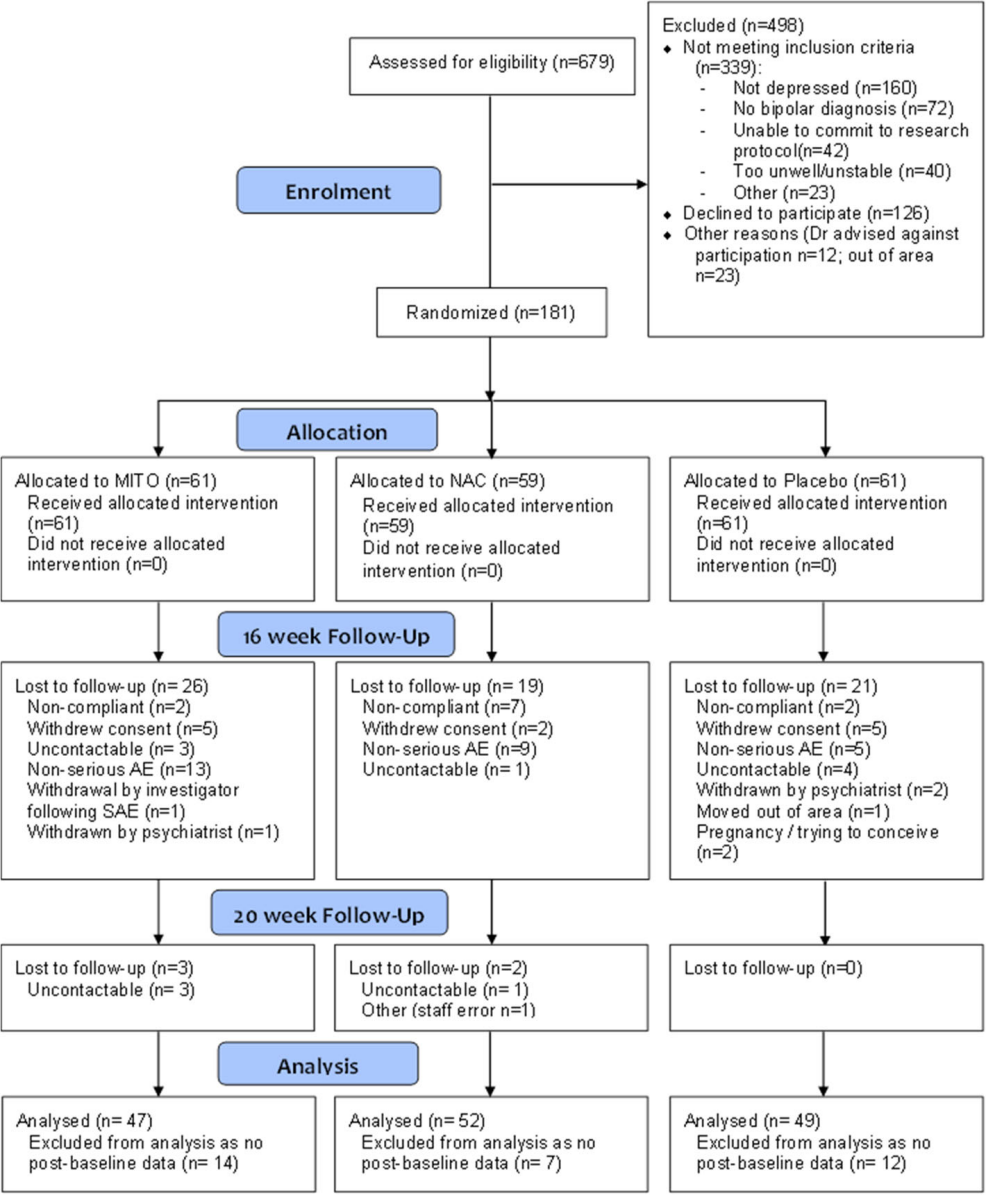
CONSORT flowchart depicting participant flow through the trial

Of the 181 cases that were randomised, 81.8% (*n* = 148) had post-randomisation data and were included for analysis (see Fig. 1). The CT had the highest proportion of cases with no post-randomisation data (23.0%, *n* = 14), followed by the placebo group (19.7%, *n* = 12) and NAC group (11.9%, *n* = 7); however, these differences between groups were not significant.

Of the 148 participants who had post-randomisation data, 77.7% (*n* = 115) had data at the primary endpoint of week 16; 81.6% (*n* = 40) in the placebo, 76.9% (*n* = 40) in NAC, and 74.5% (*n* = 35) in the CT group. The retention of participants at week 20 was also good at 73.6% (*n =* 109), with no loss of participants in the placebo group, 71.2% (*n* = 37) in the NAC, and 68.1% (*n* = 32) in the CT group. The groups did not differ significantly with respect to retention rates at weeks 16 and 20. Regarding completer analyses, there were significant differences between those who did and did not have data at the primary endpoint of week 16 and final endpoint week 20 in terms of age of diagnosis (week 16 no data *M* = 31.5, *SD* = 10.1; data *M* = 36.4, *SD =* 11.5; *t*(144) = − 2.19, *p* = .030: week 20 no data *M* = 31.8, *SD* = 9.5; data *M* = 36.6, *SD =* 11.7; *t*(144) = − 2.27, *p* = .025) and use of mood stabilisers (week 16 no data 48.5%, *n* = 16; data 74.8%, *n* = 86); *χ*^2^ (1) = 8.28, *p* = .004: week 20 no data 51.3%, *n* = 20; data 75.2%, *n* = 82; *χ*^2^ (1) = 7.69, *p* = .006).

#### Primary outcome—depressive symptoms on MADRS

**Fig. 2:**
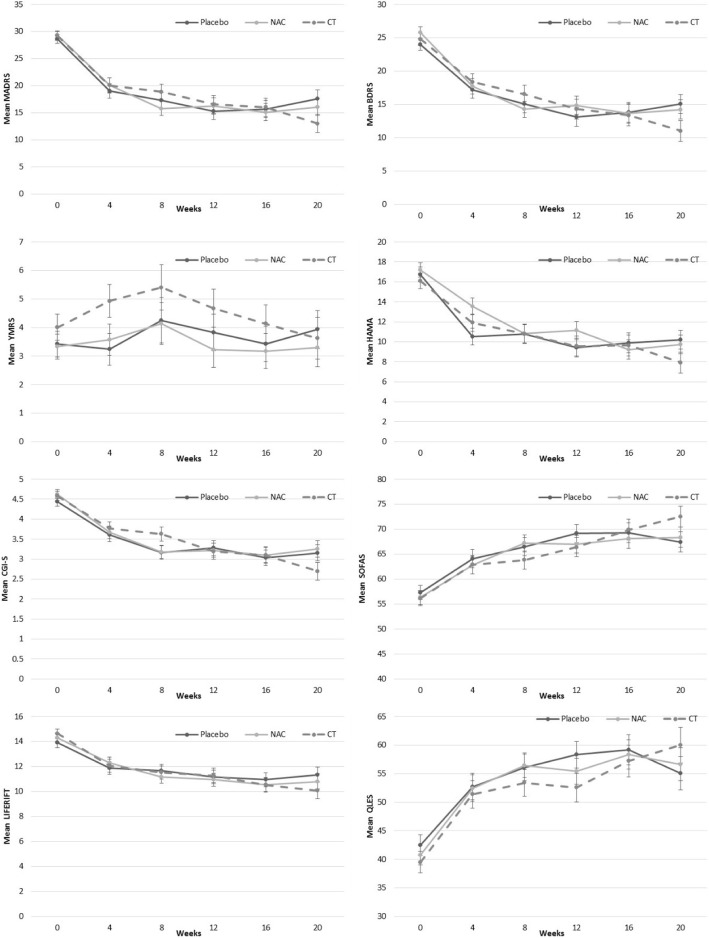
Estimated means (±SE) derived from MMRM for each of the clinical measures for the three groups and over the six time points. Note: YMRS, Young Mania Rating Scale; CGI-BP Clinical Global Impressions Scale - Bipolar; BDRS, Bipolar Depression Rating Scale; MADRS, Montgomery-Asberg Depression Rating Scale; Brief Psychiatric Rating Scale, BPRS; GAF, Global Assessment of Functioning ; SOFAS, Social and Occupational Assessment Scale; QLS, Quality of Life Scale; NAC N-acetylcysteine; CT Combination Treatment

The interaction between group and time (from baseline to week 20) for the MADRS was not significant, *F*(10, 120.8) = 1.17, *p* = .315; however, the main effect for time was significant, *F*(5, 120.9) = 74.43, *p* < .001, indicating that all groups had improvement in depressive symptoms over the duration of the trial. Examination of planned comparisons revealed that there was a significant difference between the placebo and CT groups with the CT group demonstrating a greater reduction in MADRS scores from baseline to week 20 than the placebo, (*M*_diff_ = − 5.2, *SE*_diff_ = 2.4), *t*(111.5) = − 2.19, *p = .*031 (see Fig. 2 for estimated means for each group over the time, see Table 3 for mean change scores). Post hoc comparison indicated that at the 20-week post-discontinuation visit, the CT group had a significantly lower MADRS score compared to the placebo, *p* = .046.

**Table 3:**
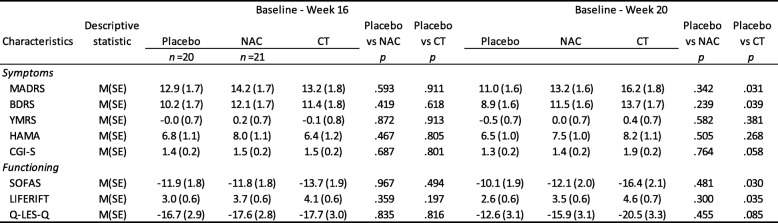
Estimated mean change (±SE) from MMRMs for baseline to week 16, and baseline to week 20, for the three groups on clinical measures

Note: YMRS, Young Mania Rating Scale; CGI-BP Clinical Global Impressions Scale - Bipolar; BDRS, Bipolar Depression Rating Scale; MADRS, Montgomery-Asberg Depression Rating Scale; Brief Psychiatric Rating Scale, BPRS; GAF, Global Assessment of Functioning ; SOFAS, Social and Occupational Assessment Scale; QLS, Quality of Life Scale

#### Secondary outcomes

The omnibus interactions for group by time for the BDRS, HAM-A, YMRS, CGI, SOFAS, LIFE-RIFT, and Q-LES-Q were all not significant; however, for all of the variables with exception of the YMRS, the time main effects in the models were significant (all *p* < .001), indicating all groups improved over time. With the planned comparisons, the rate of change between baseline and week 20 was significantly greater for the CT as compared to the placebo group for the BDRS, *M*_diff_ = − 4.82, *SE*_diff_ *=* 2.30, *t*(113.0) = 2.09, *p* = .039, the SOFAS, *M*_diff_ = 6.25, *SE*_diff_ *=* 2.84, *t* (115.9) = − 2.20, *p* = .030, and the LIFE-RIFT, *M*_diff_ = − 2.00, *SE*_diff_ *=* 0.94, *t*(120.0) = − 2.13, *p* = .035. The differences between the CT and placebo groups at 20 weeks on these variables were not significant (BDRS, *p =* .060; SOFAS, *p* = .083; LIFE-RIFT, *p* = .158). In Fig. 2, there appeared to be a separation of the groups on the YMRS in the early phase of the trial. On the YMRS, the difference between the placebo and CT groups was significantly different at the 4-week time point (*p* = .037); however, when adjusted for multiple comparisons between the groups at every time point using Bonferroni, the comparison is no longer significant (*p* = .111).

Table 4 documents differences between the three groups with respect to CGI-I and PGI-I. For the CGI-I, there was a significant difference between the placebo and CT groups at 20 weeks, *t*(106) = 2.08, *p* = .040. No other differences were noted.

**Table 4:**
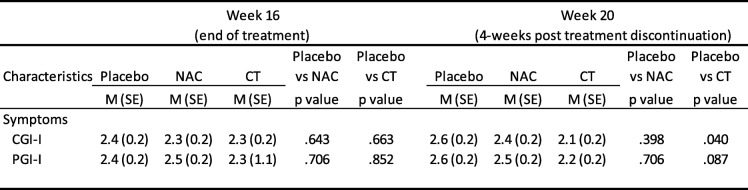
Differences between NAC and Placebo and CT versus Placebo on the clinical and patient measures of improvement

Note: CGI-I, Clinical Global Impressions - Improvement; PGI-I, Patient Global Impression - Improvement

#### Adverse effects

Adverse events (AEs) were categorised into the following categories: behavioural, gastrointestinal, musculoskeletal, neurological, respiratory, and other (see Table 5). Gastrointestinal problems were the most frequent AE noted in the cohort; 38.5% (*n* = 39) reported heartburn/reflux/indigestion, 17.8% (*n* = 18) had diarrhoea/loose stools, and 15.8% (*n* = 16) had nausea/vomiting. There were significant differences between the three groups with respect to gastrointestinal, *χ*^2^ (2) = 9.98, *p* = .007, and other AEs, *χ*^2^ (2)=7.14, *p* = .028. The number of participants reporting gastrointestinal issues in the NAC group was significantly higher than that in the placebo group, *p* < .05. The number of participants reporting other complaints was significantly higher in the placebo than in the CT group, *p* < .05. Closer examination indicated that the group difference in gastrointestinal problems was due the NAC group having significantly higher heartburn/reflux/indigestion than both other groups (both *p < .*05). No other significant differences were found.

**Table 5:** Number of participants reporting adverse events by body system

Body system category	Total	Placebo	NAC	CT
	(*N* = 181)	*n* = 61	*n* = 59	*n* = 61
Behavioural	38% (68)	33% (20)	34% (20)	44% (27)
Gastrointestinal	56% (101)	41% (25)	69% (41)*	57% (35)
Musculoskeletal	13% (24)	13% (8)	19% (11)	8% (5)
Neurological	17% (31)	23% (14)	10% (6)	18% (11)
Respiratory	34% (61)	39% (24)	36% (21)	26% (16)
Other	34% (62)	46% (28)	34% (20)	23% (14)*

Note: NAC, N-acetylcysteine; CT, combination nutraceutical treatment. * Significantly different from the placebo, all other comparisons ns

In the CT arm, 20 participants (33%) reported 26 events (17 elevation, 9 hypomania/mania); in the NAC arm, 13 participants (22%) reported 19 events (13 elevation, 6 mania/hypomania), and in the placebo arm, 14 participants (23%) reported 17 events (11 elevation, 6 hypomania/mania). Of these, four were serious AEs related to hospitalisation for mania/hypomania (2 in the placebo and 2 in the NAC groups).

## Discussion

The study provided a number of novel findings despite having an overall negative outcome as a trial. First, neither the combination of mitochondrial-modifying nutrient agents nor NAC alone separated from the placebo at the primary endpoint of the study; hence, the study is essentially negative on the primary outcome variable of symptom severity as measured by the MADRS. No significant between-group differences were observed on change scores from baseline to week 16 on any of the other clinical and functioning measures.

Second, the rate of change between baseline and week 20 post-discontinuation was significantly greater in the CT group compared with the placebo on a number of measures including depression, both BDRS and MADRS, and functioning. At the 20-week post-discontinuation, clinical improvement was significantly greater in the CT group compared to that in the placebo. This suggests either delayed benefit of the combination or an improvement of symptoms on withdrawal which warrants further exploration.

Second, the CT group had higher levels of manic symptoms at week 4 compared to the placebo. While this may reflect a type 1 error and did not survive correction for multiple comparisons, this finding provides very tentative evidence that while CT may drive mitochondrial biogenesis, this also may have the undesirable effect of worsening manic symptoms without a correspondingly robust decrease in depressive symptoms. Third, NAC alone failed to separate from the placebo on the primary mood outcome in contrast to the only previous study in bipolar disorder [7], and a number of other studies in depression, PTSD, and substance abuse [8–10]. Whether this reflects a true null effect or is a consequence of latent methodological factors remains uncertain. The rates of substance use were significantly higher in the CT group which may have adversely influenced outcomes in that group, as substance abuse predicts poorer outcomes. Critically, in the 2008 bipolar disorder study, NAC did not separate from the placebo at week 16—the endpoint of this study, and only separated at weeks 20 and 24, which suggest a delayed effect. There may be other patient or methodological differences between the studies that may explain these discordant findings. It is true both that many initially positive studies are not replicated, and equally many known efficacious agents carry the baggage of negative studies. A powerful operative factor driving null outcomes in depression studies in developed healthcare systems is “service filters”, since participants volunteering for clinical trials at tertiary treatment centres tend to self-select on the basis of treatment resistance and poor outcomes with conventional treatments and tend to be more burdened by psychological, social, and personality comorbidity.

Other methodological considerations need to be borne in mind in interpreting these data. The composition of the mitochondrial combination was based on a synthesis of the extant literature, which is by its nature, preliminary and incomplete. It is probable that more extensive pre-clinical data could result in an improved or, indeed, efficacious combination. Addition of other mitochondrially active agents such as creatine or resveratrol, or altered doses of the composition may have altered the study findings. The sample size of 181 resulting in arms with 59–61 participants each is capable of detecting moderate but not small effect sizes. In both unipolar and bipolar depression, most efficacious agents have small effect sizes, which this study may not have been able to detect. The a priori power estimate was a sample of 225, but this study recruited 181 individuals. The study duration is likely an issue. As the positive previous study of NAC was significant at the week 20 and 24 time points [7], not at the earlier week 16 time point, this study is likely to have been too short for differences to be apparent. A new study in schizophrenia separated on negative symptoms at 12 but not 6 months, suggesting that NAC is extremely slow for benefit to manifest. Those who had more stable mood stabiliser treatment were more likely to be retained in the trial. Finally, the placebo group demonstrated a mean change in the MADRS of almost 15 points. Large placebo responses are associated with a lower likelihood of showing a superiority of the active drug in clinical trials of bipolar disorder [11]. Modifiable factors associated with a higher placebo response, relevant to our study, include a lower probability of receiving the placebo and longer study duration [12, 13].

A further conclusion of the study is that the benign nature of nutraceutical approaches cannot be assumed. It is a popular view that such approaches are without risk. Yet, the possible increase in manic symptoms in this study may suggest otherwise. Any agent with neurobiological properties has the potential for these to be adverse as well as beneficial. However, the study opens the door to the utilisation of similar therapeutic strategies in other disorders characterised by impaired mitochondrial biogenesis, where the risks of mood elevation are minimal, such as chronic fatigue syndrome. Biomarker stratification has the promise of assisting with targeted therapy against identified targets, with preliminary albeit inconclusive leads to date [14].

## Conclusion

In summary, this study produced unexpected data on the potential efficacy of NAC and CT for the treatment of bipolar depression. While negative on the primary 16-week outcome, as measured by the MADRS, there was statistical separation on a number of measures at the 20-week post-discontinuation visit, which was offset by a possible increase in manic symptoms, as measured by the YMRS. Though this study does not provide a clear pathway to treat bipolar depression by augmenting mitochondrial function, it does furnish us with proof of principle and points to a mechanistic role of increased biogenesis in the pathophysiology of mania.

## Abbreviations

AE: Adverse event
ALA: Alpha lipoic acid
ALC: Acetyl l-carnitine
BDI: Bipolar disorder I
BDRS: Bipolar Depression Rating Scale
CGI: Clinical Global Impression
CGI-I: CGI-Improvement
CGI-S: CGI-Severity
CT: Combination nutraceutical treatment
HAM-A: Hamilton Anxiety Rating Scale
LIFE-RIFT: Longitudinal Interval Follow-Up Evaluation - Range of Impaired Functioning Tool
MADRS: Montgomery-Åsberg Depression Rating Scale
MMRM: Mixed model repeated measures
NAC: *N*-acetylcysteine
PGI-I: Patient Global Impression scale
Q-LES-Q-SF: Quality of Life Enjoyment and Satisfaction Questionnaire Short Form
SOFAS: Social and Occupational Functioning Assessment Scale (
YMRS: Young Mania Rating Scale
