# Correction to: A randomised controlled trial of a mitochondrial therapeutic target for bipolar depression: mitochondrial agents, N-acetylcysteine, and placebo

**DOI:** 10.1186/s12916-019-1280-2

**Published:** 2019-02-17

**Authors:** Michael Berk, Alyna Turner, Gin S. Malhi, Chee H. Ng, Susan M. Cotton, Seetal Dodd, Yuval Samuni, Michelle Tanious, Claire McAulay, Nathan Dowling, Jerome Sarris, Lauren Owen, Astrid Waterdrinker, Deidre Smith, Olivia M. Dean

**Affiliations:** 1 IMPACT Strategic Research Centre, School of Medicine, Barwon Health, Deakin University, P.O. Box 291, Geelong, VIC Australia; 2 Department of Psychiatry, University of Melbourne, Royal Melbourne Hospital, Level 1 North, Main Block, Parkville, VIC Australia; 3 Florey Institute for Neuroscience and Mental Health, University of Melbourne, Kenneth Myer Building, 30 Royal Parade, Parkville, VIC Australia; 4 Orygen, The National Centre of Excellence in Youth Mental Health, 35 Poplar Rd, Parkville, VIC Australia; 5 School of Medicine and Public Health, Faculty of Health and Medicine, the University of Newcastle, Callaghan, NSW Australia; 6 CADE Clinic, Royal North Shore Hospital, Northern Sydney Local Health District, St Leonards, NSW Australia; 7 Academic Department of Psychiatry, Kolling Institute, Northern Sydney Local Health District, St Leonards, NSW Australia; 8 ARCHI, Sydney Medical School Northern, University of Sydney, Sydney, NSW Australia; 9 Department of Psychiatry, University of Melbourne, the Melbourne Clinic, 130 Church St Richmond, Melbourne, VIC Australia; 10 Centre for Youth Mental Health, The University of Melbourne, 35 Poplar Rd, Parkville, VIC Australia; 11 NICM, School of Health and Science, Western Sydney University, Campbelltown, NSW Australia; 12 School of Psychology, University of Central Lancashire, Preston, UK; 13 Melbourne Health, 300 Grattan St, Melbourne, VIC Australia

**Correction to: BMC Med**

**https://doi.org/10.1186/s12916-019-1257-1**

The original article [1] contained two minor errors:

1. Author, Chee H. Ng’s middle initial was omitted;
2. The Competing Interests declaration omitted some information regarding author, Chee H. Ng.

These two errors have since been corrected.
